# Public health risks of humanitarian crises in Mozambique

**DOI:** 10.7189/jogh.11.03054

**Published:** 2021-03-27

**Authors:** Floriano Amimo, Anthony Magit, Jahit Sacarlal, Kenji Shibuya, Masahiro Hashizume

**Affiliations:** 1 Department of Global Health Policy, Graduate School of Medicine, The University of Tokyo, Tokyo, Japan; 2 Faculty of Medicine, Eduardo Mondlane University, Maputo, Mozambique; 3 Human Research Protection Program, University of California San Diego School of Medicine, San Diego, California, USA; 4 Institute for Population Health, King’s College London, London, UK

Mozambique has been experiencing various humanitarian crises with important public health implications over the last years. These crises compound underlying disease burden dominated by human immunodeficiency virus infection and acquired immune deficiency syndrome (HIV/AIDS), neonatal disorders, tuberculosis, and malaria [1]. Despite important health gains over the last two decades, the number of deaths and disability-adjusted life-years due to HIV/AIDS per 100 000 population increased by 1201.6% and 1034.4% from 1990 to 2019 in the country [1]. Currently, as coronavirus disease 2019 (COVID-19) responses disrupt the livelihoods of populations throughout the country, by limiting business and access to health care, thousands are being displaced due to armed conflicts in the northern and central provinces of the country, as the country heals from the consequences of recent natural disasters. Between October 2017 and October 2020, >424 200 people, mostly women and children, are estimated to have been internally displaced because of the worsening armed conflict in the northern province of Cabo Delgado [2]. Many medical staff have fled the region, leaving health facilities deserted [3]. This compounds the health risks raised by cyclones Idai and Kenneth, which on 14 March 2019 and 25 April 2019 hit the central and northern regions of the country, displacing >2.2 million people (>7.0% of country’s population) [4,5]. In the central province of Sofala, 28 out of 157 health facilities were entirely or partially destroyed [6]. The country’s health system is already fragile and underfunded: in 2015, government health spending per general government spending was 2.8%—well below the 15% mandated by Abuja Declaration (2001) [7,8]. Therefore, under these circumstances, access to essential maternal and child health products, services, and resources, including sanitation, nutrition, bed nets, antenatal care, skilled birth attendants, and childhood immunizations, becomes unattainable, risking the re-emergence of diseases that had so far been controlled, and compromising the effectiveness of efforts towards global targets. Subnationally, investment policies and provision of public services have focused more on the southern region of the country [9], resulting in important within-country geographical inequalities in the accessibility and quality of essential health products, services, and resources, further compounding the public health risks in the central and northern provinces, which account for 78.5% of country’s population [5].

**Figure 1:**
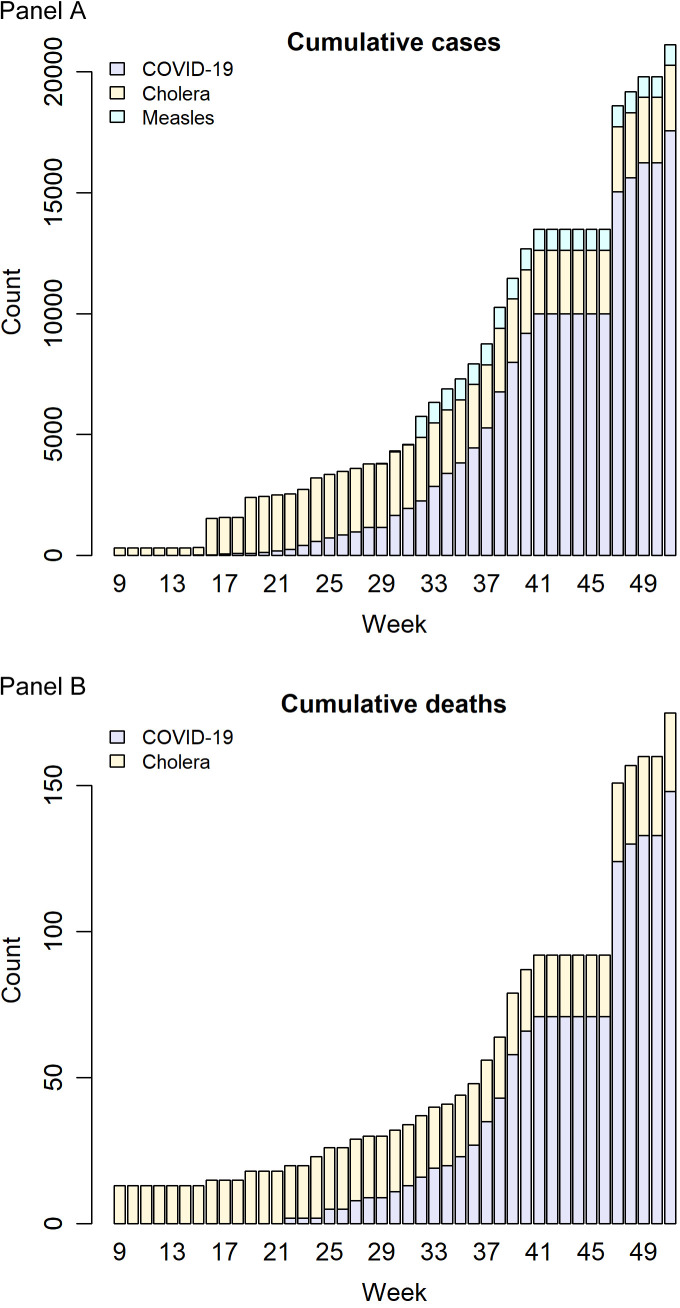
Evolution of COVID-19, measles, and cholera outbreaks in Mozambique in 2020. **Panel A.** Cumulative number of cases per disease. **Panel B.** Cumulative number of deaths per disease. Data source: WHO Regional Office for Africa weekly bulletin on outbreaks and other emergencies.

Data are limited; however, official reports have indicated that various non-COVID-19 outbreaks have been occurring in the country. Polio, cholera, and measles outbreaks have been declared in the northern and central provinces of Niassa, Cabo Delgado, Nampula, and Zambezia, as recently as January 2019, January 2020, and March 2020, respectively, with the latter outbreaks ongoing as of 20 December 2020 [10-12]. (**Figure 1**). This is despite the availability of effective MMR (measles mumps rubella) vaccine, oral cholera vaccine, and IPV (inactivated polio vaccine) in the country. The occurrence of these outbreaks, whose public health impacts might be underreported due to weak health information systems, underscores the limited performance of immunization services and global efforts in the country to effectively cover the eligible populations, which might be explained in part by populations that are difficult to reach due to frequent disasters and displacements, thus missing scheduled childhood vaccines, among other factors. As elsewhere across the continent, the response in the field is usually fragmented in the country, not only during the mentioned frequent disasters and displacements, but also in other contexts. Typically, under pressure to deliver short-term results, global health actors and programme implementers introduce parallel systems and processes [13]. In addition to its detrimental effects on health systems and aid effectiveness, there is also the risk that, under these settings, (due to fragmentation) certain health needs and/or populations might not be covered adequately, and even be neglected by not aligning with donor countries’ strategic priorities and/or domestic politics. Therefore, integrated response of the international and bilateral partners is vital to ensure that natural and manmade disasters like these do not pose significant risks to global health security (GHS), while minimizing local public health consequences. Without an effective infrastructure to protect vulnerable populations in conflict and disasters settings, the world might be at a greater risk of future resurgence of pandemics, resulting from emerging and re-emerging pathogens, as well as emergence and spread of drug-resistant pathogens.

The link between manmade and natural disasters and the emergence and spread of infectious diseases is well established. On the one hand, lack of basic needs, which characterizes post-disaster settings, may increase forest clearing and wildlife hunting, thus exposing humans more to wildlife that may serve as reservoirs for new pathogens. For example, the index patient of the 2014-16 Ebola outbreak in western Africa, reported in December 2013, was an 18-month-old boy from a small village in Guinea who is believed to have been infected by bats [14]. On the other hand, lack of drinking water, sanitation, and essential health resources, which usually follows manmade and natural disasters, may facilitate the spread of water-borne and zoonotic diseases. For example, it was among poor people, who lacked basic hygiene and sanitation infrastructure, that the Ebola outbreak had major impact in western Africa [15]. The effects of these disasters might be more severe and long-lasting in countries with fragile institutions as it is in most of Africa, since the already limited resources (including those funded/provided by international and bilateral partners, delivered to affected populations by global health actors through or working in collaboration with national/local authorities) might not reach those in need due to corruption embedded in health systems across the continent [16,17], among other factors.

Moreover, even if the ongoing COVID-19 pandemic is controlled in most of the world, the virus might persist in some locations with unstable access to essential medical products and services as it is in most of Africa, although the risks are significantly greater for countries such as Mozambique where manmade disasters are compounded by fragile institutions and high propensity to natural disasters due to geographical exposure to extreme climatic conditions and hazards such as cyclones, storms, and flash floods. In these settings, drug degradation due to inadequate storage and transport temperature is also a significant risk for the effectiveness of COVID-19 vaccination programmes.

**Figure:**
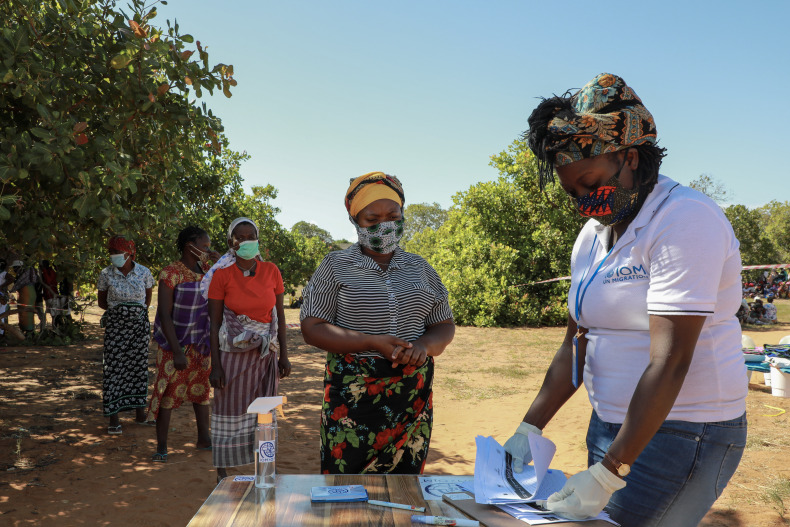
Photo: Shelter kit distribution. Source: International Organization for Migration (used with permission).

Therefore, an effective GHS infrastructure is critical. The International Health Regulations (2005) (IHR) provide an overarching legal framework that defines countries’ rights and obligations in handling public health events and emergencies that have the potential to cross borders [18]. However, this tool focuses on the response/management/surveillance of an outbreak/pandemic/health emergency. Preparedness and prevention are vital and more effective than mitigation. This means that to ensure/improve its effectiveness, IHR should be expanded to encompass evidence-based rights and obligations for countries and international partners, to improve how they manage, interact, and cooperate in disaster-settings, to minimize the risk of emergence of infectious diseases or outbreaks, instead of focusing only on how to detect, assess and report, and respond (after an outbreak/pandemic/health emergency has emerged). The effect of corruption embedded in health systems across countries with fragile institutions as well as fragmentation which characterizes global health actions in the field both need to be adequately reflected in IHR and similar frameworks if these are to be successful/effective in vulnerable countries. Embedding IHR core capacities into health system functions, strategy, and budget, at national and subnational levels, is critical to improve its effectiveness, to ensure GHS and help vulnerable countries achieve global targets [19].

The COVID-19 pandemic has shown that investing in GHS is paramount, not only for the affected populations in these settings but globally, since an outbreak that starts in one corner of the globe can easily affect all countries, disrupting trade and livelihoods globally. Investment in GHS can only be effective if it is coupled with reliable mechanisms to ensure efficiency, sustainability, fairness, and transparency, to ensure that the resources reach those in need. Therefore, without an effective GHS infrastructure, then the sustainable development goals (SDGs) [20], which are part of the United Nations’ resolution 70/1 – Transforming our world: The 2030 Agenda for Sustainable Development – might not be achieved in most of the continent, and the world will remain at a significant risk of future pandemics. Under the current setup, there is a significant risk that given the frequent disasters and displacements, the SDGs target 3.8 – achieve universal health coverage, including financial risk protection, access to quality essential health care services, and access to safe, effective, quality, and affordable essential medicines and vaccines for all – might not be achieved in the country, particularly in the central and northern provinces. As a result, other SDGs, including target 3.3 – by 2030, end the epidemics of AIDS, tuberculosis, malaria and neglected tropical diseases and combat hepatitis, water-borne diseases and other communicable diseases – might not be achieved as well.
